# Epidemiological investigation of venomous snakebites in Yunnan Province

**DOI:** 10.3389/ftox.2025.1609487

**Published:** 2025-08-06

**Authors:** Qinfen Gao, Yajun Teng, Chao Xiao, Rui Zeng, Bin Han, Hong Gao, Jianhai Wang, Xiaoyan Li, Canju Yang, Jianneng Dai, Chunxi Li, Qunyan Huang, Zengzheng Li, Wei Zhang

**Affiliations:** ^1^Department of Emergency, The First People’s Hospital of Yunnan Province, The Affiliated Hospital of Kunming University of Science and Technology, Kunming, China; ^2^Department of Emergency, Guangnan County People’s Hospital, Wenshan, China; ^3^Department of Emergency, Lincang Second People’s Hospital, Lincang, China; ^4^Department of Emergency, Qujing City first People’s Hospital, Qujing, China; ^5^Department of Emergency, The First Affiliated Hospital of Dali University, Dali, China; ^6^Department of Hematology, Baoshan People’s Hospital, Baoshan, China; ^7^Department of Emergency, Dali People’s Hospital, Dali, China; ^8^Department of Emergency, Honghe State first People’s Hospital, Mengzi, China; ^9^Department of Emergency, Xishuangbanna People’s Hospital, Jinghong, China; ^10^Department of Emergency, Xuanwei First People’s Hospital, Xuanwei, China; ^11^Department of Hematology, The First People’s Hospital of Yunnan Province, The Affiliated Hospital of Kunming University of Science and Technology, Kunming, China; ^12^ Department of Critical Care Medicine, The First Affiliated Hospital of Kunming Medical College, Kunming, China

**Keywords:** venomous snakebite, epidemiology, treatment, Yunnan Province, snakebite management

## Abstract

**Background:**

Snakebite envenoming constitutes a substantial public health concern worldwide. Yunnan Province, The lack of comprehensive epidemiological data on snakebite in Yunnan affects research, diagnostic, and treatment advancements. This research evaluates patient demographics, seasonal patterns, snake species associated with the disease, and treatment approaches to guide preventative and therapeutic initiatives in the province.

**Methods:**

This retrospective analysis reviewed clinical records of venomous snakebite cases admitted to hospitals in 16 cities within Yunnan Province from January 2022 to November 2024. Collected data covered diverse aspects, including patient demographics (age, sex), circumstances of the bite (location, size, and time), species identification, observed clinical symptoms, treatments administered (e.g., antivenin and alternative therapies), and hospital stay duration. Subsequently, the effect of different therapeutic measures on these patients’ hospital stays was analyzed.

**Results:**

A total of 2,112 venomous snakebite cases were recorded, with incidence rates rising annually: 406/46.73 million in 2022, 825/46.73 million in 2023, and 886/46.73 million in 2024. *Ovophis* (52.08%) and *Trimeresurus* (28.74%) species were predominant. These findings align with the annual distribution of anti-venom serum administered to affected individuals. Most incidents occurred during June to September, primarily in mountainous and forested areas or paddy fields, comprising approximately 52.40% of the total cases. Nearly all bites (99.05%) were localized to the limbs, presenting with swelling and pain as the dominant clinical features. Statistical analysis revealed that factors such as incision and debridement, additional anti-venom serum, fibrinogen supplementation, plasma administration, Ji Desheng Snake Medicine, and magnesium sulfate compresses were significantly associated with extended hospital stays (*P* < 0.05).

**Conclusion:**

Snake bites in Yunnan Province mainly affect young and middle-aged agricultural workers in rural and mountainous areas. The predominant venomous snakes in the area are hemotoxic. The findings emphasize the necessity of early intervention with antivenom and adjunctive therapies, including fibrinogen and plasma administration. Delays in getting medical help or improper treatment can lead to longer hospital stays.

## 1 Introduction

Snakebite envenoming (SBE) is a severe medical condition with significant social and economic impacts. Globally, 4.5 to 5.4 million snakebite cases occur each year, with mortality rates comparable to those of drug-resistant tuberculosis and multiple myeloma (Alcoba et al., 2022; Afroz et al., 2024). Moreover, the number of amputations and permanent disabilities caused by SBE is three times higher than the number of deaths. Severe envenoming results in about 400,000 cases of permanent disability and causes between 81,000 and 138,000 deaths annually (Alcoba et al., 2022; Afroz et al., 2024; Ahmed et al., 2008).

The burden of SBE is excessively high in developing regions, with the greatest incidence reported in East Asia, South-East Asia, and sub-Saharan Africa (Alirol et al., 2010). Vulnerable groups include women, children, and agricultural workers in underprivileged rural communities of low- and middle-income countries (Ahmed et al., 2008; Kasturiratne et al., 2008). For instance, China experiences an estimated 250,000 to 280,000 snakebite cases annually, with mortality rates ranging from 5% to 10% and a disability rate affecting 25%–30% of survivors (Liang et al., 2022). Recognizing its significant public health impact, the World Health Organization (WHO) designated SBE as a neglected tropical disease (NTD) in 2009. It elevated its status to Category A NTD in 2017 (GBD 2019 Snakebite Envenomation Collaborators, 2022). The WHO seeks to achieve a 50% reduction in snakebite-related mortality and disability by the year 2030 (Minghui et al., 2019). Achieving this goal requires comprehensive epidemiological studies in endemic regions to inform effective prevention and control strategies.

In China, the absence of a comprehensive epidemiological surveillance and reporting system results in a considerable underreporting of snakebite incidents (Hu et al., 2023). Surveys have reported that over 60 species of venomous snakes inhabit China, with snakebite occurrences peaking during the summer and autumn months (April-October) (Lai et al., 2024). In the southeastern part of China, namely Hangzhou (Shen et al., 2024), and in the northwestern part of the country, namely Guizhou (Gou et al., 2024), as well as in southern provinces such as Guangdong (Tu et al., 2025) and Guangxi (Liang et al., 2022), epidemiological investigations on snakebite incidents have been conducted. However, there are still no statistical data on the incidence rates of snakebites in each province. Yunnan Province, located in the southwestern border region of China, is characterized by various climates, including tropical, subtropical, temperate, and cold zones. This diverse landscape and rich vegetation support a remarkable variety of biodiversity. As a result, Yunnan has a particularly high incidence of snakebites and is also characterized by ethnic diversity and high poverty levels (Zhong et al., 2015). Young workers often suffer from snakebites, and when affected by venomous species, the rates of disability and mortality are significantly increased, imposing significant expenses on families and society. Therefore, there is a critical need for comprehensive, high-quality multi-center epidemiological studies.

The present study aims to provide a detailed analysis of venomous snakebite epidemiology in Yunnan Province over the past 3 years, focusing on case distribution, identification of high-risk groups, seasonal variations, prevalent venomous species, and treatment strategies. The findings from this study will serve as a valuable resource for developing effective prevention and control strategies for venomous snakebites in Yunnan and across China, ultimately contributing to public health improvements. Furthermore, this research will enrich the theoretical and practical knowledge base regarding epidemiological studies on venomous snakebites in China.

## 2 Materials and methods

### 2.1 Ethics statement

This study was approved by the Medical Ethics Committee of the First People’s Hospital of Yunnan Province. Ethics number: KHLL2024-KY087.

### 2.2 Study settings

This study employed a retrospective analysis method to collect and organize the data of venomous snake bite cases from the emergency departments of 36 medical institutions in 16 cities and counties within Yunnan Province from January 2022 to November 2024. The data were retrieved through the hospitals’ information systems, including the medical record front page and case query systems, and all the medical staff of these 36 medical institutions were members of the Yunnan Snake Bite Research Collaboration Group (YSBSRCG). Inclusion criteria included: (1) a confirmed snakebite history, (2) the snake body or photographs of a venomous snake presented by the patient or witness, and (3) patients bitten by unidentified organisms, whose bites were assessed and confirmed by attending or senior physicians as venomous snake based on clinical evaluation, or by providing the Chinese Venomous Snake Identification Guide for the patient to identify. If the coagulation function indicates VICC, it is judged as a venomous snake of the hemotoxic type. If the necrosis of tissues is the main feature, it is a cytotoxic type of venomous snake. If the patient shows flaccid and descending paralysis, it is a neurotoxic type of venomous snake. Exclusion criteria included (1) patients with incomplete data and (2) patients with “dry bites” or without a confirmed snakebite history who required only outpatient observation and treatment. A total of 2,112 patients were included in the analysis, and data were compiled from their medical histories, clinical symptoms, diagnostic findings, and treatment records. The collected data covered patient demographics (age, gender), bite details (location, time, venomous snake species), clinical symptoms (e.g., swelling, bleeding, tissue necrosis, numbness, dizziness, palpitations, chest tightness, weakness, and dyspnea), treatment protocols, and hospital stay duration. The main clinical symptoms recorded were swelling, bleeding, tissue necrosis, numbness, and systemic symptoms such as dizziness, palpitations, chest tightness, weakness, and dyspnea. The treatment protocols included local therapy (e.g., incision, debridement, local blocking, external application of JiDeshengg Snake Medicine, and wet compresses with magnesium sulfate) and systemic treatment (e.g., anti-venom serum, plasma, fibrinogen, tranexamic acid, and oral JiDeshengg Snake Medicine), with the time of patient recovery and discharge also documented. Finally, a literature review was conducted to obtain relevant epidemiological studies on snakebites in China and internationally, which enhanced the depth of the analysis.

### 2.3 Statistical methods

The natural environment and representative snake species in Yunnan Province were statistically analyzed. Mapping was performed using ArcGIS software, while SPSS was used to process the patients’ basic data. A normality test was applied to determine the data’s distribution, with an independent samples T-test used for datasets with a normal distribution. The Mann-Whitney U test was applied to compare two groups for non-normally distributed datasets. The data were categorized, and Spearman’s rank correlation coefficient was used to analyze the relationships between variables. GraphPad Prism 9 was used to present the results graphically. Using logistic regression analysis to predict the factors that influence the length of hospital stay for patients with venomous snakebite-induced blood and venomous snakes belonging to the blood toxin category.

## 3 Results

### 3.1 The distribution of snakebite cases in each region of Yunnan Province

**FIGURE 1:**
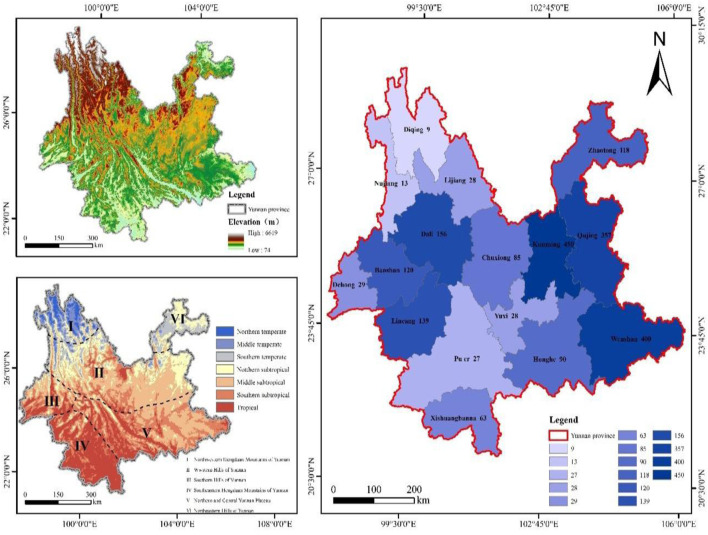
The geographical characteristics of Yunnan Province and the quantity of venomous snakebites in diverse regions.

The distribution of snakebite cases across Yunnan Province is closely associated with the province’s climatic conditions, topography, and the variety of snake species inhabiting different areas. The province is divided into six distinct regions based on these factors: The Northwestern Hengduan Mountains, the Western Hills, the Southern Hills, the Southeastern Hills, the Northern and Central Yunnan Plateau, and the Northeastern Hills, as depicted in Figure 1. Each region differs in its temperature, altitude, and the dominant snake species found there, as summarized in Table 1. The common venomous snakes in Yunnan include: Ovophis, Trimeresurus, Agkistrodon, Anilius, Ophiophagus, Naja, Vipera, etc. Ovophis, Trimeresurus, and Agkistrodon are all venomous snakes belonging to the blood toxin category. They mainly cause coagulation disorders, known as venom-induced consumptive coagulopathy (VICC), characterized by bleeding at the wound site. In severe cases, important organs may also bleed. The Anilius genus and Ophiophagus species are neurotoxic snakes, mainly presenting with flaccid and descending paralysis. Severe cases may experience bleeding, breathing difficulties, and even secondary respiratory arrest. The Naja species are cytotoxic snakes, mainly exhibiting swelling, pain, blisters, skin necrosis or infection. In severe cases, bone and fascial compartment syndrome may occur. Vipera snakes possess both blood toxins and renal cell toxins (Lai et al., 2024). This regional classification is based on updated data provided by experts from the Yunnan Academy of Animal Sciences, which includes a comprehensive analysis of Yunnan’s reptile fauna and its geographical divisions (Wang et al., 2022). The number of venomous snakebite cases diagnosed and treated over 3 years in several prefectural-level cities is as follows: Kunming City (450 cases), Wenshan Zhuang and Miao Autonomous Prefecture (400 cases), Qujing City (357 cases), Dali Bai Autonomous Prefecture (156 cases), Lincang City (139 cases), Baoshan City (120 cases), Zhaotong City (118 cases), Honghe Hani and Yi Autonomous Prefecture (90 cases), Chuxiong Yi Autonomous Prefecture (85 cases), Xishuangbanna Dai Autonomous Prefecture (63 cases), Dehong Dai and Jingpo Autonomous Prefecture (29 cases), Yuxi City (28 cases), Lijiang City (28 cases), Pu’er City (27 cases), Nujiang Lisu Autonomous Prefecture (13 cases), and Diqing Tibetan Autonomous Prefecture (9 cases).

**TABLE 1:** The descriptions of different regions in Yunnan Province and their representative snake species.

	Ⅰ	Ⅱ	Ⅲ	Ⅳ	Ⅴ	Ⅵ
	Northwestern Hengduan Mountains of Yunnan	Western Hills of Yunnan	Southern Hills of Yunnan	Southeastern Hills of Yunnan	Northern and Central Yunnan Plateau	Northeastern Hills of Yun
The principal administrative divisions	The Diqing Tibetan Autonomous Prefecture, Lijiang City, and the northern portion of the Dali Bai Autonomous Prefecture	The Nujiang Lisu Autonomous Prefecture, the Dehong Dai and Jingpo Autonomous Prefecture, Baoshan City, the southwestern area of the Dali Bai Autonomous Prefecture and Lincang City	Pu’er City, Xishuangbana Dai Autonomous Prefecture	The southern region of Yuxi City, the majority of Honghe Hani and Yi Autonomous Prefecture, the northern area of Pu’er City, the southern section of Chuxiong Yi Autonomous Prefecture, and Wenshan Zhuang and Miao Autonomous Prefecture	The central and eastern areas of Dali City, the central and northern regions of Chuxiong Yi Autonomous Prefecture, Kunming City, the southern section of Zhaotong City, Yuxi City, Qujing City, and the northern portion of Honghe Hani and Yi Autonomous Prefecture	The majority of the areas of Zhaotong City, with the exception of Qiaojia County
Regional representative snake species	Thermophis shangrila, Protobothrops xiang- chengensis, Lycodon serratus, Gloydius monti-cola	Trimeresurus yingjiangensis, Oligodon hamptoni, Rhabdophis himalayanus, Ovophis zayuensis	Chrysopelea paradoxa, Dendrelaphis vogeli, Pareasa menglaensis, Rhabdophis siamensis, Xenopeltis unicolor, Parafimbrios lao, Trimeresurus guoi	Achalinus pingbianensis, Sinonatrix percarinata jingdongensis, Calamaria yunnanensis	Oligodon yunnanensis, Amphiesma octolineatum, Amphiesma stenotaeniatum, Lycodon aulicus dongchuanensis	Achalinus spinalis, Achalinus meiguensis, Dinodon rufozonatum, Rhabdophis tigrinus
Viperidae	Agkistrodon strauchii, Ovophis monticola, Protobothrops jerdonii, Trimeresurus stejnegeri, Protobothrops xiangchengensis, *Trimeresurus albolabris*	Ovophis monticola, Protobothrops jerdonii, Protobothrops mucrosquamatus, *Trimeresurus albolabris*, Trimeresurus yunnanensis	Ovophis monticola, Protobothrops jerdonii, *Trimeresurus albolabris*, Trimeresurus stejnegeri fujianensis, Trimeresurus stejnegeri yunnanensis	Protobothrops Azemiops kharini, mucrosquamatus, *Trimeresurus albolabris*, Trimeresurus yunnanensis, Trimeresurus stejnegeri fujianensis	Ovophis monticola, Protobothrops jerdonii, Protobothrops mucrosquamatus, Trimeresurus stejnegeri fujianensis, Trimeresurus stejnegeri yunnanensis	Ovophis monticola, Protobothrops jerdonii, *Trimeresurus albolabris*, Trimeresurus stejnegeri fujianensis, Trimeresurus stejnegeri yunnanensis

### 3.2 Common venomous snake species

**FIGURE 2:**
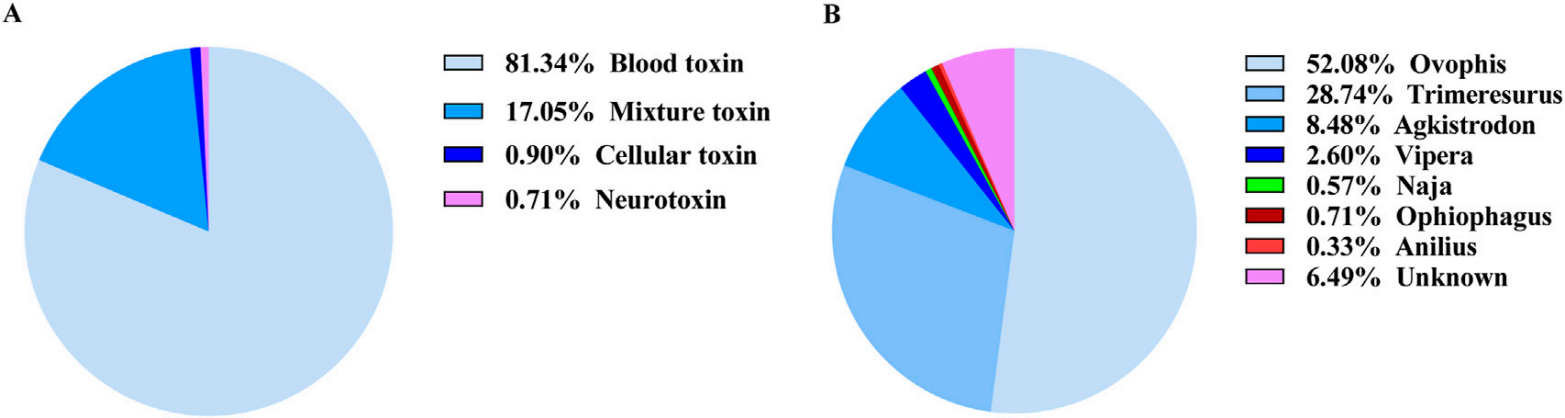
The composition of venomous snakebites in Yunnan Province within the cohort. **(A)** The proportion of snakebites by different toxins, **(B)** The proportion of snakebites by different genera of venomous snakes.

**FIGURE 3:**
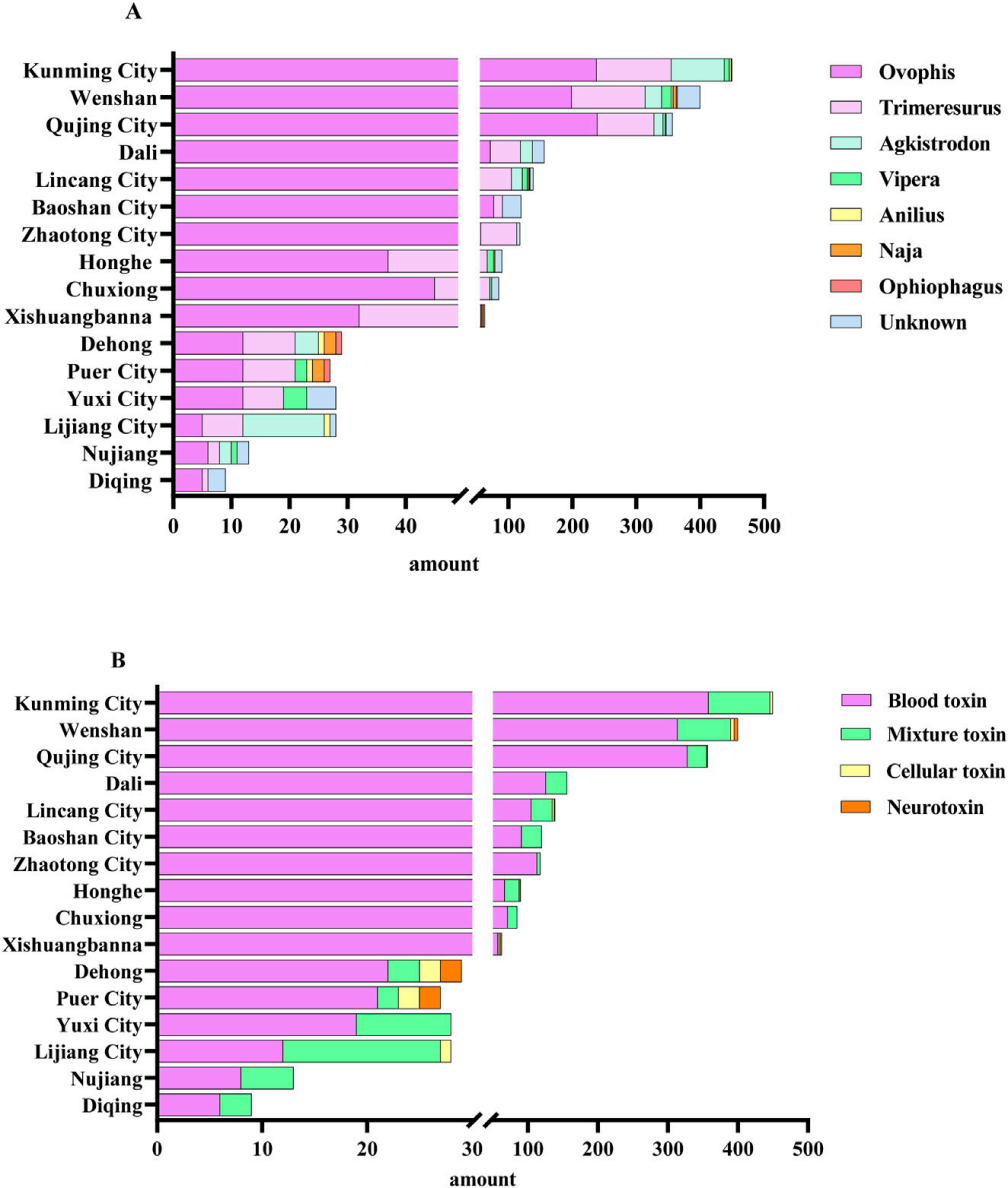
The proportion of venomous snakebite cases in various regions of Yunnan Province within the cohort. **(A)** The proportion of snakebite cases by different genera of venomous snakes, **(B)** The proportion of snakebite cases by different types of venom.

According to the latest Chinese Snakebite Treatment Guidelines, in clinical practice, snakebites are classified based on the toxic effects of snake venom on the human body into neurotoxic, hemotoxic, and cytotoxic types. When compiling statistics, those snakes with multiple toxic effects are collectively referred to as mixed toxins (Lai et al., 2024). Based on the case data from different regions, the snakes were counted and statistically analyzed. The analysis revealed that hemotoxicity constituted 81.34% of cases, mixed toxicity 17.05%, cytotoxicity 0.90%, and neurotoxicity 0.71%, as depicted in Figure 2A. Taxonomic classification of snake genera highlighted the dominance of the genus Ovophis at 52.08%, followed by *Trimeresurus* (28.74%), *Agkistrodon* (8.48%), *Vipera* (2.60%), *Naja* (0.57%), *Anilius* (0.33%), and *Ophiophagus* (0.71%), as illustrated in Figure 2B. Further, evidence corresponding to the characteristics of non-venomous snakes constituted 6.49% of the total findings (Figure 2). Venomous snake species were categorized and summarized across various regions based on geographic distribution. Within the prefectures and cities of Yunnan Province, hemotoxic venomous snakes belonging to the genera Ovophis and *Trimeresurus* were most prevalent. In Lincang City, Honghe Prefecture, Xishuangbanna Prefecture, Pu’er City, and Wenshan Prefecture, neurotoxic and cytotoxic venomous species, including those of the genera *Naja*, *Anilius*, and *Ophiophagus*, were identified. These findings are comprehensively presented in Figure 3 and Tables 2, 3.

**TABLE 2:** Statistical data of bite cases by different genera of venomous snakes in various regions of yunnan province (cases).

	Ovophis	Trimeresurus	Agkistrodon	Vipera	Anilius	Naja	Ophiophagus	Unknown	total
Diqing	5	1	0	0	0	0	0	3	9
Nujiang	6	2	2	1	0	0	0	2	13
Lijiang City	5	7	14	0	1	0	0	1	28
Yuxi City	12	7	0	4	0	0	0	5	28
Puer City	12	9	0	2	1	2	1	0	27
Dehong	12	9	4	0	1	2	1	0	29
Xishuangbanna	32	25	0	0	1	2	1	2	63
Chuxiong	45	26	0	3	0	0	0	11	85
Honghe	37	30	0	10	0	2	0	11	90
Zhaotong City	57	56	0	0	0	0	0	5	118
Baoshan City	77	14	0	0	0	0	0	29	120
Lincang City	52	53	17	8	2	1	1	5	139
Dali	72	47	19	0	0	0	0	18	156
Qujing City	239	89	14	4	0	1	0	10	357
Wenshan	199	115	26	15	3	5	2	35	400
Kunming City	238	117	83	8	3	0	1	0	450
total	1,100	607	179	55	12	15	7	137	2,112

**TABLE 3:** Statistical data of bite cases by different toxin snake genera in various regions of yunnan province (cases).

	Blood toxin	Mixture toxin	Cellular toxin	Neurotoxin	Total
Diqing	6	3	0	0	9
Nujiang	8	5	0	0	13
Lijiang City	12	15	1	0	28
Yuxi City	19	9	0	0	28
Puer City	21	2	2	2	27
Dehong	22	3	2	2	29
Xishuangbanna	57	2	2	2	63
Chuxiong	71	14	0	0	85
Honghe	67	21	0	2	90
Zhaotong City	113	5	0	0	118
Baoshan City	91	29	0	0	120
Lincang City	105	30	3	1	139
Dali	126	30	0	0	156
Qujing City	328	28	0	1	357
Wenshan	314	76	5	5	400
Kunming City	358	88	4	0	450

### 3.3 The time distribution of snakebites

**FIGURE 4:**
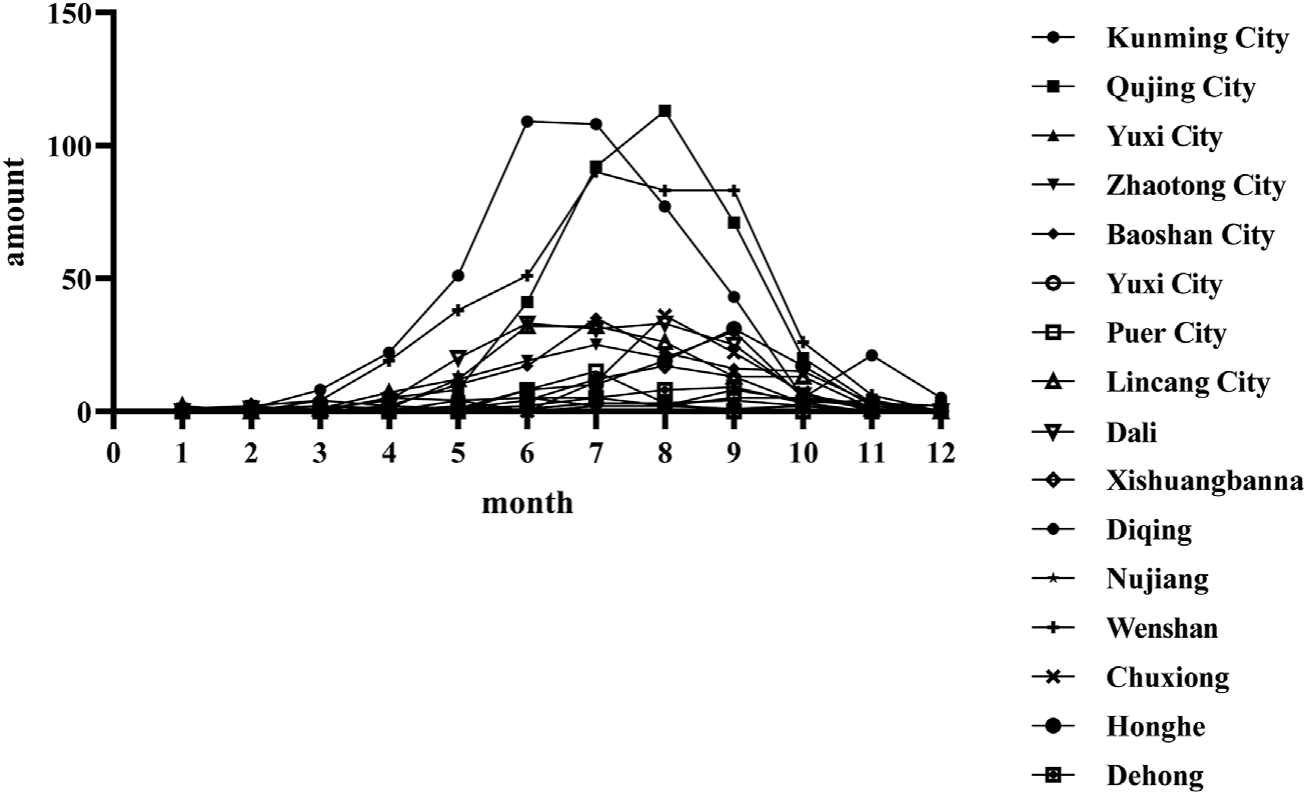
The incidence of venomous snakebite cases in yunnan province at different times.

The timing of patient visits was statistically analyzed to evaluate snakes’ seasonal activity patterns. Snakebite incidents were predominantly reported between May and October, with the highest number of cases recorded from June to September. Moreover, a few instances persisted into November, specifically in Kunming, the provincial capital (Figure 4).

### 3.4 Spatial distribution of snake bites

**FIGURE 5:**
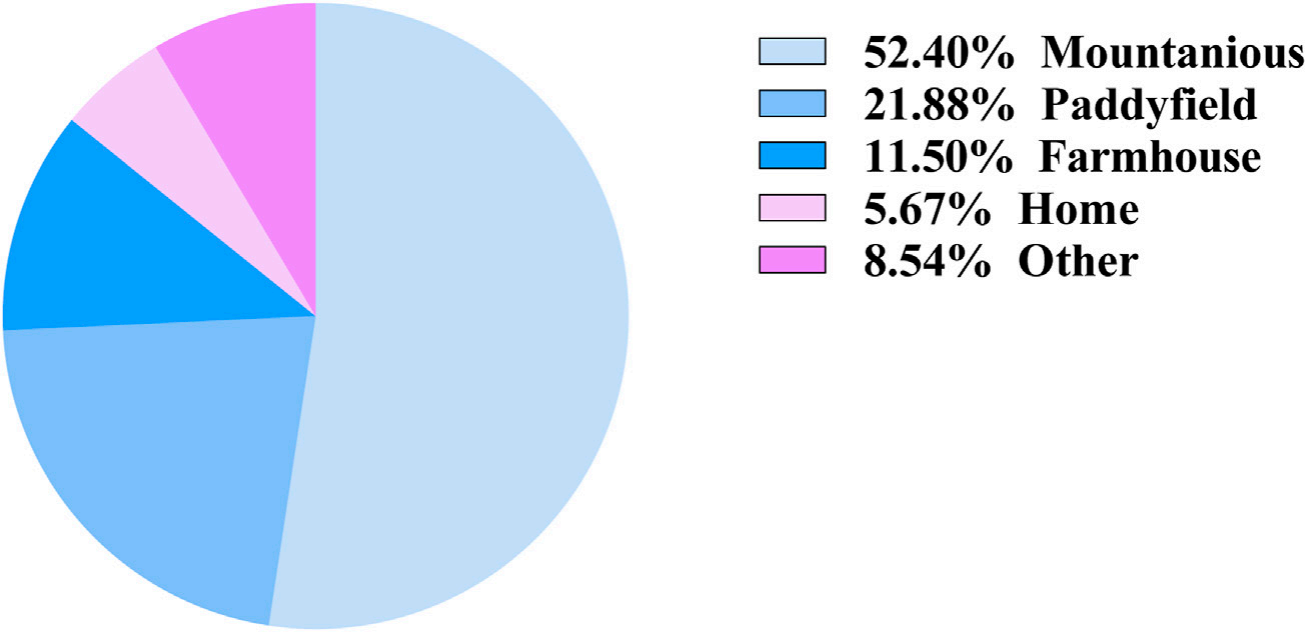
The circumstances of venomous snakebite occurrence locations in yunnan province.

The distribution of injury locations among patients was statistically analyzed. The primary injury sites were mountain forests or mountainous regions, representing 52.4% of the cases, followed by paddy fields (21.88%), areas near farmhouses (11.5%), homes (5.67%), and other less common locations (6.54%) (Figure 5). These findings may be explained by the natural habitat of snakes and the predominantly mountainous terrain of Yunnan.

### 3.5 Age characteristics of snake bites

**FIGURE 6:**
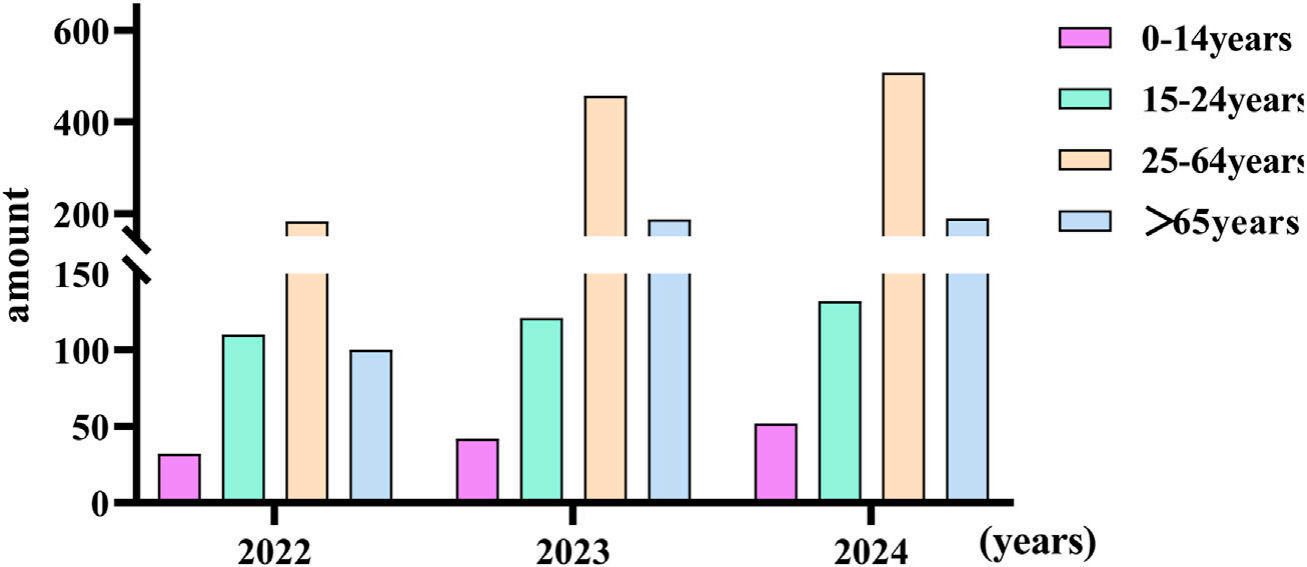
The situation of venomous snakebite in different age groups in yunnan province.

**FIGURE 7:**
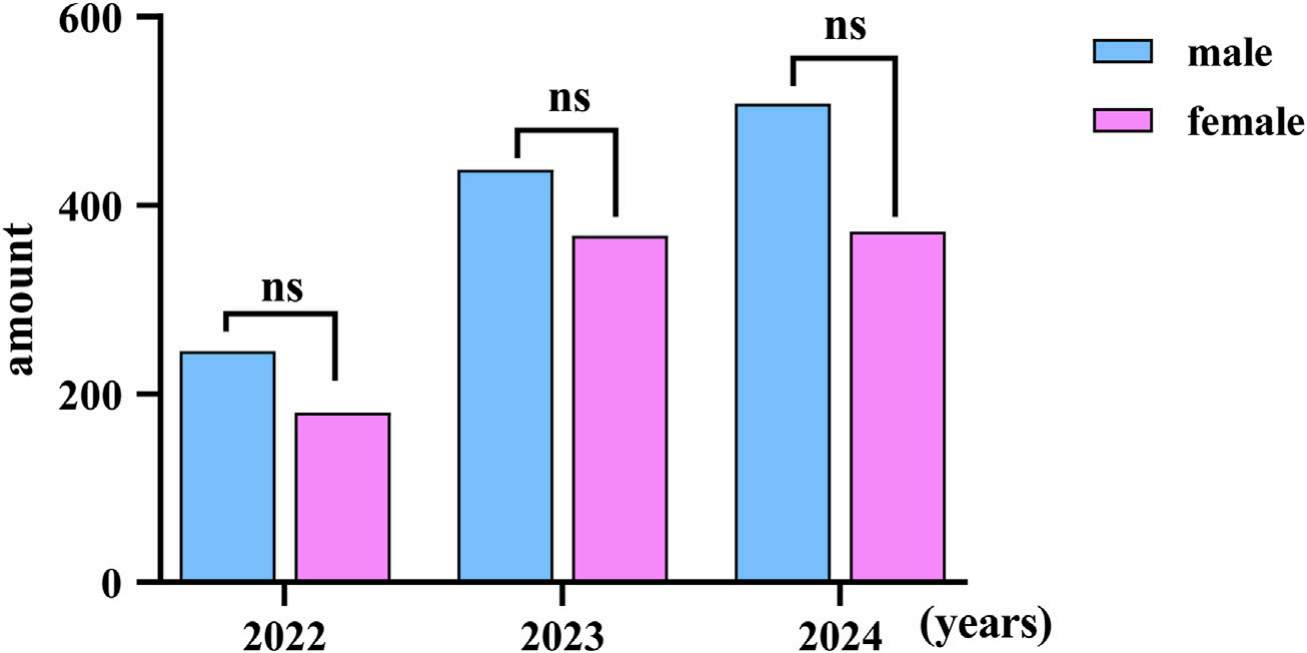
The discrepancies of venomous snakebite between different genders in Yunnan Province; ns: no statistical difference.

According to the new age classification standard of the World Health Organization, it is divided into four different age groups: Children: 0–14 years. Youth: 15–24 years. Adults: 25–64 years. Seniors/Older Persons: 65 years and over (Ahmad et al., 2001). The subjects to be included are divided into four different groups. Most snakebite patients occur among adults. (Figure 6). There was no significant difference in the number of male and female patients bitten by venomous snakes (*P* > 0.05) (Figure 7). The significant increase in the number of injuries in 2023 and 2024 was related to the enhanced awareness of those bitten to seek medical treatment, as well as the establishment of the snake-bite treatment network and database in Yunnan Province. There was no statistically significant difference (*P* > 0.05).

### 3.6 Characteristics of the bite site of venomous snakes

**FIGURE 8:**
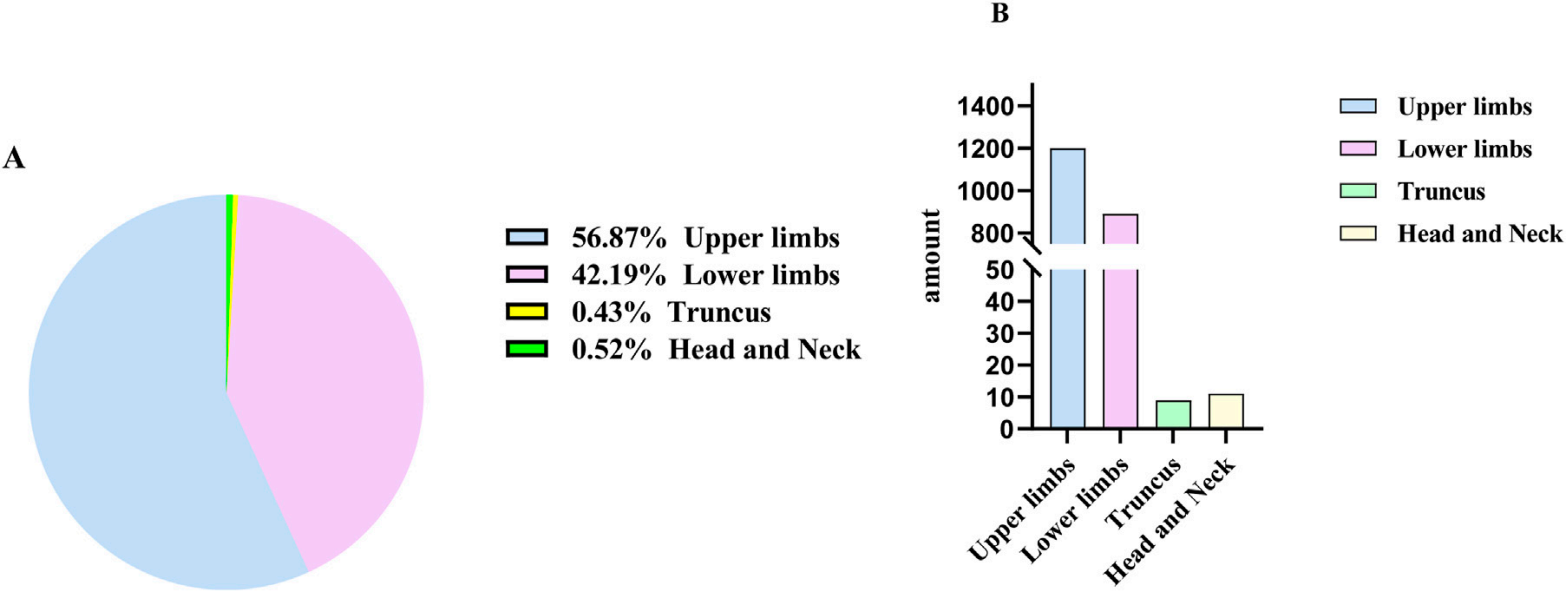
The Circumstance of Snakebite Locations in Yunnan Province, Divided into percentage **(A)** and amount **(B)**.

An analysis of the provided case histories for screening revealed that 56.87% of patient injuries occurred in the upper limbs, 42.19% occurred in the lower limbs. Head and neck injuries followed at 0.52%, while injuries to the trunk were the least common at 0.43% (Figure 8). Since the data did not follow a normal distribution, we used the Mann-Whitney U test to conduct a difference analysis of the data. The result showed that there was no statistically significant difference among the various injured areas (P > 0.05).

### 3.7 Clinical features of venomous snakebite

**FIGURE 9:**
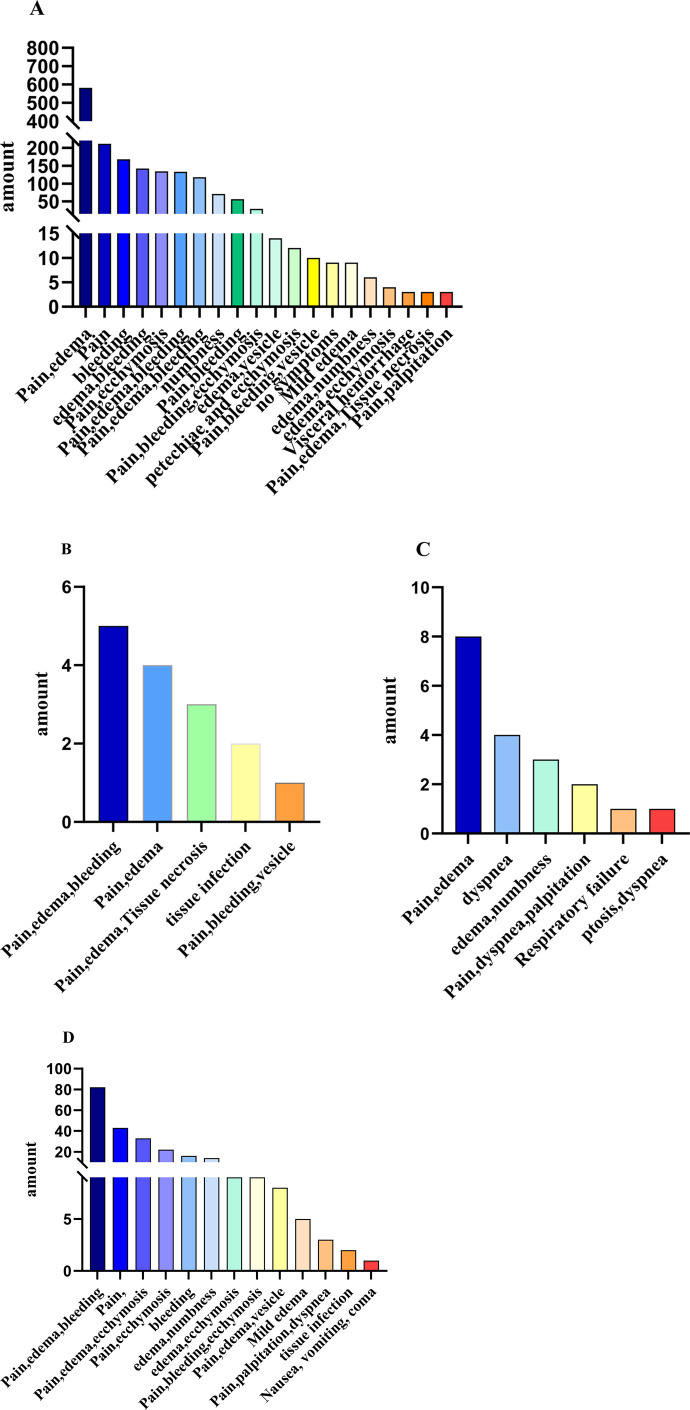
The number of cases with main clinical manifestations after bites by different types of venomous snakes. **(A)** Hemotoxic, **(B)** Cytotoxic, **(C)** Neurotoxic, **(D)** Mixed Toxic.

The clinical symptom classification outlined in the 2024 Chinese Guidelines for Snakebite Treatment (Lai et al., 2024), the clinical symptoms of patients in this study were categorized into general, hematological, cytotoxic, and neurological manifestations. General symptoms included bite marks, nausea, vomiting, fatigue, abdominal pain, dizziness, headache, fever, sweating, diarrhea, hypotension, and in severe cases, shock. Hematological symptoms were characterized by wound bleeding, petechiae, ecchymoses, hematemesis, melena, hematuria, and occasionally, bleeding in internal organs. Cytotoxic symptoms encompass swelling, pain, blister formation, local necrosis or infection, ecchymosis, paresthesia, limb paralysis, and compartment syndrome-like presentations. Neurological symptoms included bilateral ptosis, dysphagia, respiratory distress, and acute respiratory failure. The clinical manifestations varied based on the type of venom secreted by different snake species. Hemotoxic venom primarily caused pain and tissue swelling (585/1720, 34.01%) and was associated with subcutaneous hemorrhage (168/1720, 9.77%) and internal organ bleeding (3/1720, 0.17%) (Figure 9A). Cytotoxic venom also resulted in pain and swelling (5/15, 33.33%), alongside tissue necrosis (3/5.20%) and infection (2/5, 13.33%) (Figure 9B). Neurotoxic venom predominantly caused pain and swelling (8/19, 42.11%), with a subset of patients showing severe symptoms such as breathing difficulty (4/19, 21.05%), respiratory failure (1/19, 5.26%), and ptosis (1/19, 5.26%) (Figure 9C). Mixed venom presented with pain and swelling (111/358, 31.01%) and a broader range of systemic symptoms, including nausea, vomiting, palpitations, and coma (Figure 9D).

### 3.8 UsagThe time of administering the first dose of antivenom serume of antivenom serum

**FIGURE 10:**
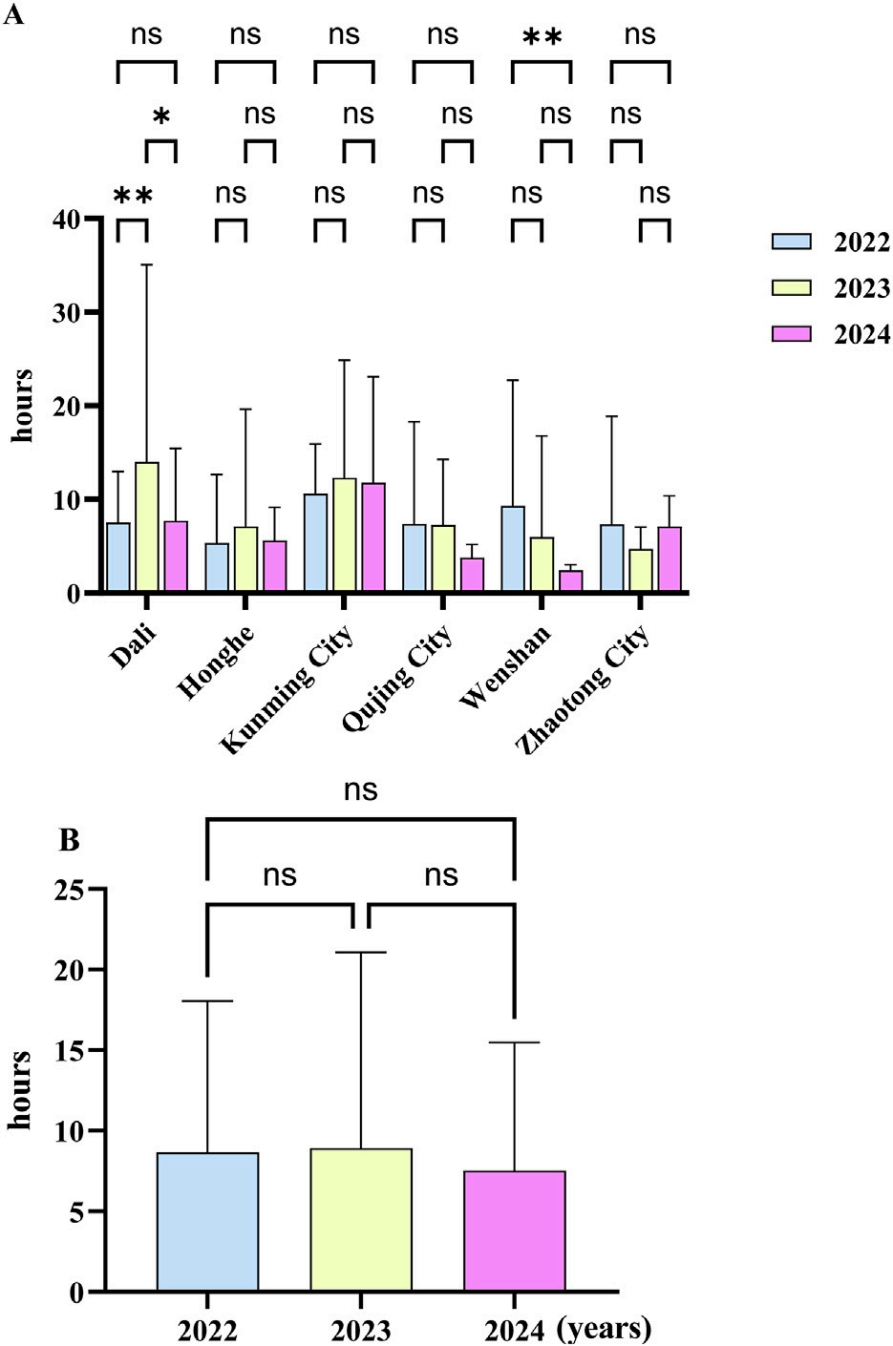
The execution time of the first antivenom serum prescription after snakebite occurred in yunnan province from 2022 to 2024. **(A)** Mean ± standard deviation of the execution time in different regions; **(B)** mean ± standard deviation of the execution time in different years in yunnan province. (*: P < 0.05, **: P < 0.005; ns: P > 0.05).

The time from the patient’s arrival at the hospital after the bite to the first administration of antivenom serum was statistically analyzed (Table 4). At the same time, a variance analysis was conducted to compare the differences in the time of the first injection of antivenom serum after snakebite in the six regions over the past 3 years. The results showed that there were significant differences between 2022 and 2023, and between 2023 and 2024 in Dali, and between 2022 and 2024 in Wenshan. The results are shown in Figure 10A. There was no statistically significant difference between 2022 and 2024 (P > 0.05) (Figure 10B). In this study, we were pleasantly surprised to find that in the past 3 years, the time interval between the snakebite and the administration of the first dose of antivenom serum in some areas of Yunnan Province has gradually shortened. As shown in Figure 10, in most areas, patients reached the hospital and received the antivenom injection within 10 h after being bitten. This might be an important reason why no deaths due to snakebite were observed in our study.

**TABLE 4:** The time of the first injection of antivenom serum after snakebite in each state is presented as the mean ± standard deviation.

	The time of the first serum injection					
	Dali	Honghe	Kunming	Qujing	Wenshan	Zhaotong
2022	7.46 ± 0.77	5.42 ± 2.01	10.58 ± 0.59	7.44 ± 1.20	9.37 ± 1.81	7.83 ± 2.68
2023	14.02 ± 2.74	7.13 ± 1.98	12.31 ± 1.30	7.29 ± 1.08	5.99 ± 1.26	4.72 ± 0.54
2024	7.73 ± 1.22	5.61 ± 0.74	11.78 ± 1.49	3.77 ± 0.39	2.44 ± 0.09	7.13 ± 0.53

### 3.9 Usage of antivenom serum

**FIGURE 11:**
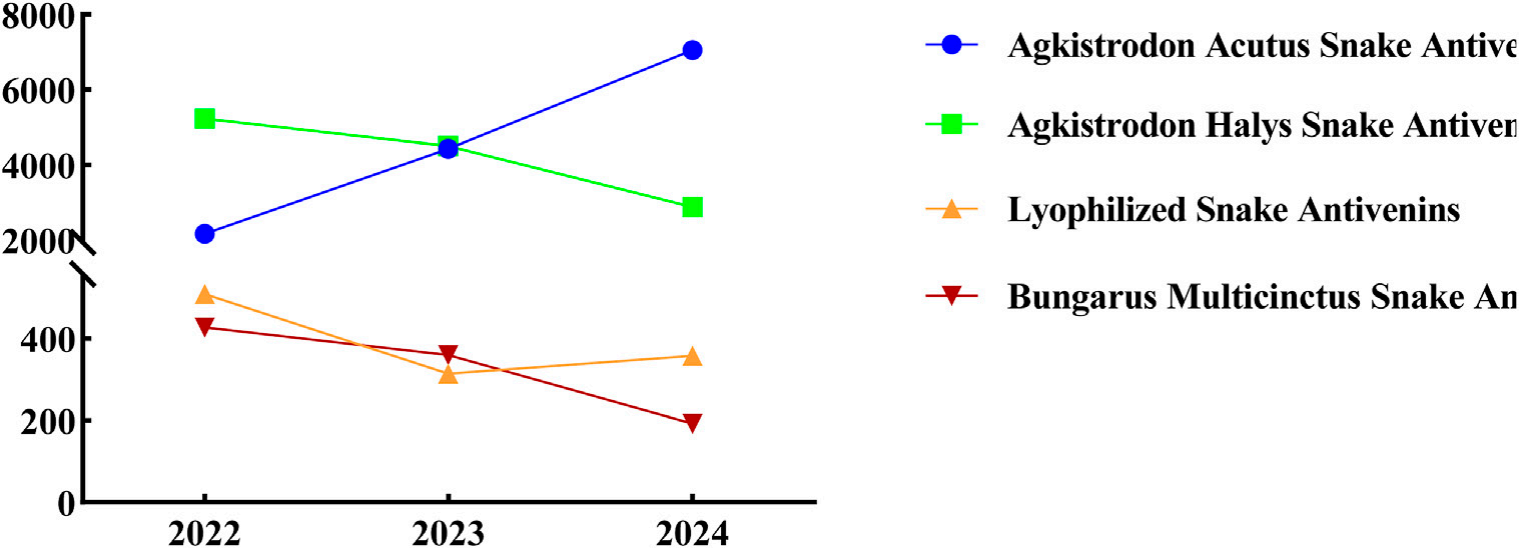
Utilization of different antivenom serums.

With the improvement of the snake bite treatment network in Yunnan Province, each snake bite treatment sub-center in Yunnan has reserves of four types of single-price antivenom sera. The Chinese Snake Bite Diagnosis and Treatment Guidelines (Lai et al., 2024) state that for bites by the green vine snake or the original pit viper, antivenom serum against the five-step snake should be used first, followed by antivenom serum against the viper or combined medication; for viper bites, antivenom serum against the five-step snake and antivenom serum against the viper should be used; for the bite of the eye-catching king snake, antivenom serum against the silver ring snake and antivenom serum against the eye-catching snake should be used, and sufficient antivenom serum against the silver ring snake should be used first; for the bite of the golden ring snake, antivenom serum against the silver ring snake should be used; for the bite of the sea snake, antivenom serum against the silver ring snake and antivenom serum against the eye-catching snake should be used in combination. If the snake species is unclear, the selection should be based on the clinical poisoning manifestations, such as using antivenom serum against the silver ring snake for neurotoxic snake bites, choosing antivenom serum against the five-step snake and/or antivenom serum against the viper for blood-toxic snake bites, and selecting antivenom serum against the eye-catching snake and/or antivenom serum against the five-step snake for cytotoxic snake bites. Therefore, the species of the venomous snake can be inferred based on the use of antivenom sera. The administration of Agkistrodon acutus antivenin has consistently risen over the years, whereas the application of Agkistrodon halys antivenin has progressively decreased. In comparis on, the use of lyophilized and *Bungarus multicinctus* snake antivenins has remained relatively low (Figure 11). These findings further suggest that the venomous snakes found in Yunnan Province are predominantly hemotoxic.

### 3.10 Other treatment measures

**FIGURE 12:**
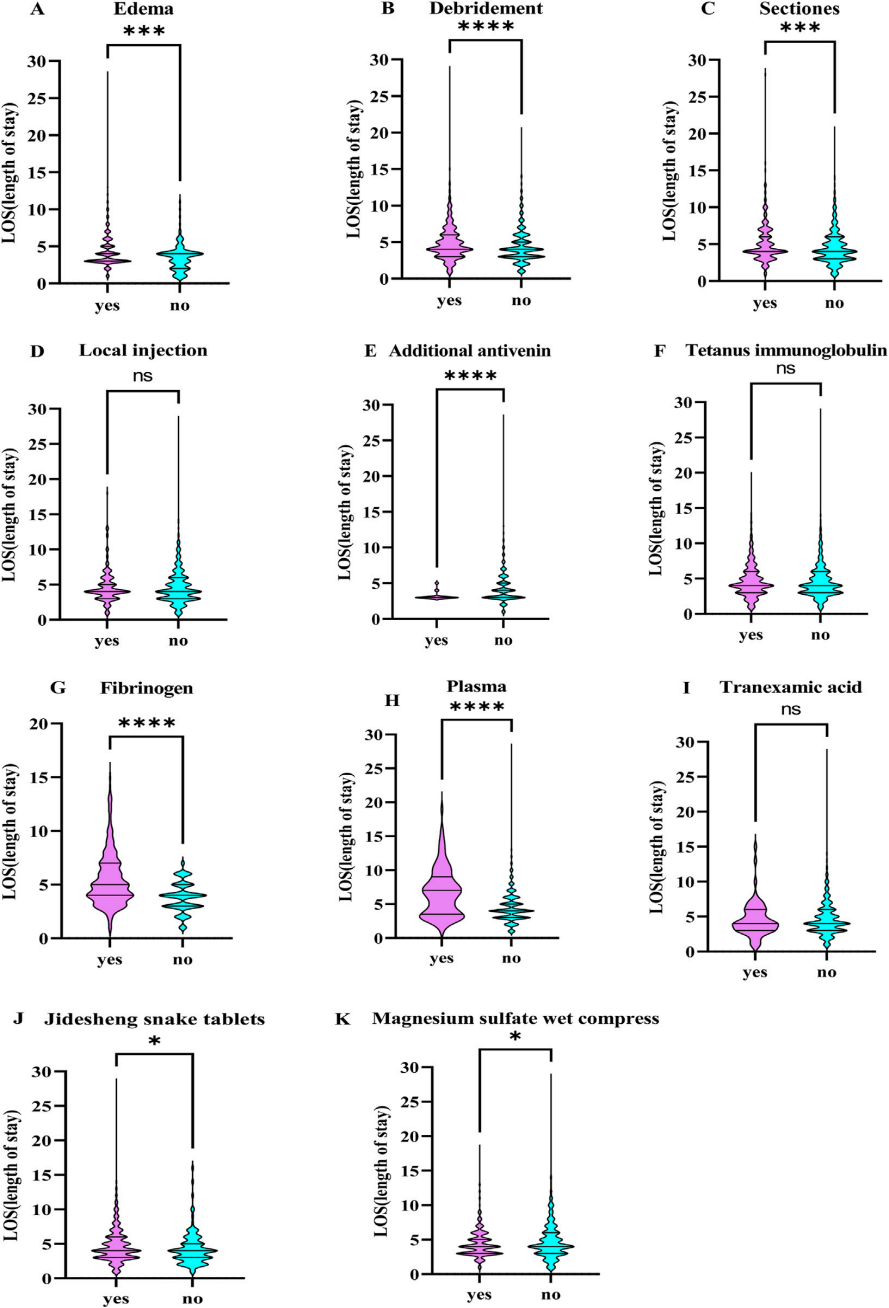
The Correlation between Different Treatment Approaches and the Length of Hospital Stay. **(A)** Edema, **(B)** Debridemnent, **(C)** Sectiones, **(D)** Local injection, **(E)** Additional antivenin, **(F)** Tetanus imununoglob ulin, **(G)** Fibrinogen, **(H)** Plasina, **(I)** Tranexamnic acid, **(J)** Jidlesheng snake tablets, **(K)** Magnesiumn sulfate wet comnpress. *: *P*<0.05, **: *P*<0.01, ***: *P*<0.001, ****: *P*<0.0001, ns: There was no statistically significant difference.

In this study, the length of hospitalization was used in this investigation as an indicator to assess the effectiveness of different treatments. The study evaluated the association between several factors, including tissue swelling, incision, debridement, local closure, supplementary antivenom therapy, tetanus immunoglobulin treatment, plasma and fibrinogen infusions, tranexamic acid, JiDesheng snake drug treatment, and magnesium sulfate compresses, with the length of the hospitalization. Significant correlations were found between hospitalization duration and tissue swelling, incision, debridement, additional antivenom treatment, plasma and fibrinogen infusions, JiDesheng snake therapy, and magnesium sulfate compresses (*P* < 0.05), as depicted in Table 5 and Figure 12.

**TABLE 5:** The therapeutic conditions of the patient during hospitalization.

Group		LOS(length of stay)	Z	P
Edema	yes (n = 1919)	4 (3, 5)	−3.517	<0.001^a^
	no (n = 128)	4 (2, 4)		
Debridement	yes (n = 727)	4 (3, 6)	−5.278	<0.001^a^
	no (n = 826)	4 (3, 5)		
Sectiones	yes (n = 256)	4 (4, 6)	−3.886	<0.001^a^
	no (n = 1,297)	4 (3, 6)		
Local injection	yes (n = 248)	4 (3, 5)	−0.72	0.472
	no (n = 1,305)	4 (3, 6)		
Additional antivenin	yes (n = 76)	3 (3, 3)	−5.283	<0.001^a^
	no (n = 1912)	4 (3, 5)		
Tetanus immunoglobulin	yes (n = 794)	4 (3, 6)	−1.286	0.198
	no (n = 753)	4 (3, 6)		
Fibrinogen	yes (n = 229)	5 (4, 7)	−6.499	<0.001^a^
	no (n = 1,321)	4 (3, 5)		
Plasma	yes (n = 101)	7 (3.5, 9)	−6.458	<0.001^a^
	no (n = 1,451)	4 (3, 5)		
Tranexamic acid	yes (n = 63)	4 (3, 6)	−0.192	0.848
	no (n = 1,489)	4 (3, 6)		
JiDesheng snake tablets	yes (n = 1,379)	4 (3, 6)	−2.033	0.042
	no (n = 115)	4 (3, 5)		
Magnesium sulfate wet compress	yes (n = 555)	4 (3, 5)	−2.042	0.041
	no (n = 992)	4 (3, 6)		

We used the Mann-Whitney U test to conduct a difference analysis of the data.

^a^ Indicates P < 0.05, indicating a statistically significant difference.

### 3.11 Analysis of factors affecting hospital stay duration

According to the research reports, we divided the hospital stay time into less than or equal to 3 days and more than 3 days (Aga et al., 2025). Using logistic binary regression analysis, it was found that debridement (OR = 0.501, 95% CI [0.366–0.368]) and supplementing fibrinogen (OR = 0.360, 95% CI [0.224–0.580]) might be beneficial for shortening the patient’s hospital stay, especially the use of fibrinogen was more significant (Table 6), while the use of tranexamic acid was not conducive to shortening the hospital stay; for patients with non-blood toxic snake bites, local incision (OR = 0.324, 95% CI [0.126–0.834]) might shorten the patient’s hospital stay (Table 7).

**TABLE 6:** Logistic regression analysis of risk factors affecting hospitalization time in patients bitten by Venomous snakes belonging to the blood toxin category.

	β	S.E	Chi-square value	p-value	OR	95%CI for OR
Debridement	−0.691	0.160	18.591	0.000^a^	0.501	0.366–0.686
Sectiones	0.093	0.129	0.525	0.469	1.098	0.853–1.413
Local injection	0.080	0.208	0.150	0.699	1.084	0.721–1.628
Tetanus immunoglobulin	0.090	0.149	0.369	0.544	1.095	0.818–1.465
Fibrinogen	−1.021	0.243	17.640	0.000^a^	0.360	0.224–0.580
Plasma	−0.522	0.293	3.179	0.075	0.593	0.334–1.053
Tranexamic acid	0.775	0.364	4.537	0.033^a^	2.171	1.064–4.430
JiDesheng snake tablets	−0.274	0.239	1.315	0.251	0.760	0.476–1.215
Magnesium sulfate wet compress	0.007	0.142	0.002	0.962	1.007	0.762–1.330

Using logistic regression analysis to predict the factors influencing the length of hospital stay for patients with venomous snakes belonging to the blood toxin categor.

^a^ Indicates P < 0.05.

**TABLE 7:** Logistic regression analysis of risk factors affecting hospitalization time in patients bitten by Non-blood-toxin venomous snakes.

	β	S.E	Chi-square value	p-value	OR	95%CI for OR
Debridement	−0.165	0.291	0.320	0.572	0.848	0.479–1.501
Sectiones	−1.128	0.483	5.453	0.020^a^	0.324	0.126–0.834
Local injection	0.027	0.322	0.007	0.934	1.027	0.547–1.929
Tetanus immunoglobulin	0.171	0.287	0.356	0.551	1.187	0.676–2.085
JiDesheng snake tablets	−0.473	0.423	1.250	0.264	0.623	0.272–1.428
Magnesium sulfate wet compress	0.212	0.251	0.711	0.399	1.236	0.755–2.022

Using logistic regression analysis to predict the factors influencing the length of hospital stay for patients with Non-blood-toxin venomous snakes.

^a^ Indicates P < 0.05.

## 4 Discussion

Yunnan Province is located in the southwestern part of China, with longitudes between 97°31′and 106°12′east and latitudes ranging from 21°08′to 29°15′north. The province is divided into 16 prefectures and 128 counties, including five county-level districts. Known for its mountainous plateau, Yunnan has a subtropical plateau monsoon climate (Zhu et al., 2016). The highest point in the province is Meili Snow Mountain in northwest Yunnan, reaching an elevation of 6,740 m, while the lowest point is in Hekou County, southeast Yunnan, at 76.4 m above sea level. Although Yunnan is located in a low-latitude region, its diverse terrain and significant elevation variations result in a highly varied climate. This variation gives rise to different climatic zones in China and globally, including tropical, subtropical, temperate, and cold zones (Hua, 2012). The province’s vegetation can be classified into three main types: tropical rainforest, subtropical evergreen broadleaf forest, and subalpine coniferous forest (Chen et al., 2024). Yunnan typically experiences a small annual temperature range with significant daily fluctuations. The average yearly temperature ranges from 4.7°C to 23.7°C. This climatic and ecological diversity creates a suitable environment for a wide range of snake species (Chen et al., 2021) (Figure 1). The peak seasons for snakebites generally occur during the summer and autumn months, characterized by higher temperatures, increased humidity, and increased snake activity. In the present study, venomous snakebites were most frequently reported between June and September. This period coincides with the busy farming season, during which agricultural production and labor activities intensify, resulting in a higher frequency of snakebites, particularly among farmers. However, in recent years, improvements in urban greening have led to an increased snake population in urban areas, thus raising the number of snakebites during citizens’ outdoor activities. This finding is consistent with similar studies conducted in other regions (Seifert and Armitage, 2022). Furthermore, the provincial capital, Kunming, experiences a peak in snakebites in November. This is likely due to Kunming’s spring-like climate year-round, which allows snake activity to remain consistent. Furthermore, the increased awareness and educational efforts among the public may also contribute to this peak. Kunming, the primary snakebite treatment center in Yunnan Province, offers comprehensive medical services, including a full range of antivenoms, specialized treatment facilities, and accessible transportation. As a result, many patients from various districts and counties travel to Kunming for diagnosis and treatment.

In an 8-year epidemiological study of snakebites in Kashan, Iran (2004–2011), the majority of snakebite victims were male (96%). Age distribution showed that the highest incidence of snakebites occurred in the 15–24-year-old age group (Dehghani et al., 2012). Similarly, a study involving 1,500 snakebite cases at a tertiary care center in India found that 65% of the victims were male, with 48% aged between 21 and 40. The analysis suggested that these individuals were mostly engaged in outdoor activities related to agriculture (Kumar et al., 2018). However, in the present study, the victims were predominantly young and middle-aged adults ranging from 25 to 64 years. The majority of injuries occurred in mountainous forests (52.4%) and rice paddies (21.88%), with most injuries affecting the extremities (99.05%). These patterns might be correlated with factors such as the economy, geography, the working population, and work habits in Yunnan Province. The terrain of the province is predominantly mountainous, and its population relies heavily on modern agriculture suited to the plateau environment. Young and middle-aged individuals work in fields or mountains throughout the year to meet their obligations. Further, the lack of protective measures during these activities makes them particularly vulnerable to snakebites. Based on these findings, strengthening public awareness campaigns is recommended. During peak snake activity seasons, outdoor activities should be minimized, and training programs for high-risk groups should be expanded. Workers are also advised to wear long-sleeve clothing, long trousers, rain boots, and gloves when working in areas where snakes may be present (Vaiyapuri et al., 2022). Furthermore, carrying snakebite antivenom could be a precautionary measure in an emergency.

China is widely recognized as having one of the highest levels of snake species diversity globally, with over 300 documented species, including more than 100 venomous ones (Lai et al., 2024). In Yunnan Province alone, 147 snake species have been identified across 15 families and 50 genera (Hou et al., 2021; Li et al., 2020). Clinically, snake venoms are categorized into neurotoxic, hemotoxic, cytotoxic, and mixed-toxic types based on their specific physiological effects. Some venomous species may produce a combination of toxins. For instance, the Ophiophagus primarily contains neurotoxins and hemotoxins, while the Agkistrodon halys, although primarily hemotoxic, also produces neurotoxins (Lai et al., 2024). The high diversity of venomous snake species in Yunnan Province presents challenges in species identification due to morphological similarities among many species. Thus, patients often provide imprecise or incomplete descriptions of the snakes involved in their envenomations. Physicians primarily rely on clinical presentations, wound characteristics, and laboratory diagnostics to infer the species responsible for the bite. Furthermore, due to fear or limited knowledge, patients may fail to provide critical evidence, such as snake remnants, which complicates identification. As a result, species classification is typically based on the venom’s toxicological profile and the genus of the snake. These findings indicate that the majority of venomous snakebites in Yunnan Province are attributed to blood-venomous snakes of the genera *Ovophis* (52.08%) and *Trimeresurus* (28.74%), followed by *Agkistrodon* (8.48%), *Vipera* (2.60%), *Naja* (0.57%), *Anilius* (0.33%), and *Ophiophagus* (0.71%). This distribution aligns with the significant use of antivenom serum for *Bungarus multicinctus* and *Agkistrodon acutus*, as shown in Figure 9. In remote areas of Yunnan, particularly in mountainous regions, health institutions such as township health centers lack sufficient antivenom stocks. Many county-level hospitals possess only a single type of antivenom serum, which is inadequate to address the diverse needs of snakebite victims, often delaying timely treatment. Patients may also experience prolonged travel times to medical facilities due to challenging terrain and poor transportation. Based on the investigation, it is recommended that basic health facilities in remote areas be equipped with antivenom serum, particularly for hemotoxic snake species. In regions such as Lincang City, Honghe Prefecture, Xishuangbanna Prefecture, Pu’er City, and Wenshan Prefecture, where species such as *Naja*, *Bungarus*, and *Ophiophagus* are prevalent, it is essential to stock antivenom for *B. multicinctus* and *Naja atra* to improve treatment success rates (Iliyasu et al., 2015).

The clinical manifestations following venomous snakebites can be categorized into three distinct groups based on the nature of the venom: the neurotoxic triad (characterized by bilateral ptosis, descending paralysis, and dyspnea/acute respiratory failure), the hematotoxic triad (involving venom-induced consumptive coagulopathy, local hemorrhage, and systemic hemorrhage), and the cytotoxic triad (featuring severe pain, progressive swelling, and tissue damage) (Seifert and Armitage, 2022). In the current study, limb pain and swelling (33.90%) were the predominant clinical symptoms observed, with subcutaneous hemorrhage occurring infrequently. Although most bites occurred on the hands or feet, swelling frequently extended to the trunk. In cases of severe swelling, hypotension may develop (Hifumi et al., 2013). Furthermore, data revealed that 81.34% of the snakebites were attributable to hemotoxic snakes. According to foreign studies (Chan et al., 2016), venom from these snakes typically contains coagulation toxins and other substances such as hemolytic toxins, hemorrhagic toxins, fibrinogen, phospholipase A2, and active protein C (APC) inhibitors. The thrombin-like enzyme in the venom shows activity similar to thrombin, facilitating the generation of fibrin monomers and activating the fibrinolytic system. The combined action of fibrinolytic enzymes in the venom induces defibrinogenemia, a condition known as DIC, which is part of a broader disorder known as venom-induced consumptive coagulopathy (VICC) (Si et al., 2014). VICC is predominantly characterized by bleeding, and in severe cases, it can progress to a non-coagulating blood state, resulting in continuous bleeding from wounds, hematuria, gastrointestinal hemorrhage, and even hemothorax and intracranial bleeding. This condition is associated with a high mortality rate (Tan et al., 2021; Maduwage and Isbister, 2014), demanding urgent clinical attention.

In the latest Chinese guidelines for treating snake bites, it is stated that antivenom serum is the only safe and effective drug against snake venom, and it follows the principles of timely and adequate administration, timely supplementation, use of the same species, and combination of different species. (Lai et al., 2024). Antivenoms neutralize venom by binding to it, and both early administration and proper dosing significantly influence patient outcomes. Reducing the time between envenomation and the first dose of antivenom is critical for improving prognosis (Isbister, 2023). In this study, we were pleasantly surprised to find that in the past 3 years, the time interval between the snakebite and the administration of the first dose of antivenom serum in some areas of Yunnan Province has gradually shortened. As shown in Figure 10, in most areas, patients reached the hospital and received the antivenom injection within 10 h after being bitten. This might be an important reason why no deaths due to snakebite were observed in our study. As shown in Figure 10A, regions such as Dali, Honghe, Kunming, Qujing, and Wenshan have experienced a substantial reduction in the time for the first dose of antivenom in recent years, indicating a significant improvement in awareness and access to timely care. However, areas such as Zhaotong, Diqing, and Nujiang, situated at higher altitudes in Yunnan’s northeastern, northern, and northwestern parts, report fewer snakebite cases. According to observation, These regions face challenges related to limited economic development and medical resources, which hinder effective snakebite treatment. Enhancing professional training for healthcare providers in these areas is necessary to improve treatment standards. Several regions in the southwestern and southeastern parts of Yunnan, including Pu’er, Lincang, Baoshan, Chuxiong, Dehong, Honghe, and Xishuangbanna, experience a higher incidence of snakebites due to their favorable climate and proximity to Myanmar. However, data reporting in these areas remains insufficient, which complicates the assessment of snakebite management. Many individuals who suffer from snakebites experience extreme financial challenges, preventing them from affording the high costs associated with antivenom serum and subsequently avoiding its administration. These circumstances contribute to incomplete and difficult-to-interpret statistical data regarding snakebite incidents. To address these issues, there is an urgent need to strengthen the infrastructure of information systems, broaden the coverage of snakebite-related data networks, and improve public awareness. Moreover, it is critical to enhance the clinical capabilities of pre-hospital emergency personnel and medical professionals in snakebite management (Margono et al., 2022), refine medical insurance policies for antivenom serum, and minimize the delay in administering antivenom to improve patient outcomes and increase the overall cure rate. According to the pricing contract with the producer of antivenom serum antivenin against *A. acutus* venom 1,128/RMB/branch, antivenin against *Agkistrodon halys* venom 1,128/RMB/branch, antivenin against *N. atra* venom 2,500/RMB/branch, antivenin against *B. multicinctus* venom 1,400/RMB/branch. According to the survey, in 2020, the net income of families with government subsidies was 17,170.00 RMB, and the net income of families without government subsidies was 20,910.00 RMB (Liu, 2024). Currently, there is still a lack of evidence regarding the ideal dose of the first anti-snake venom serum. Clinicians can flexibly determine the dose based on the type of snake, regional differences, severity, and time of visit. Combined with the experience of using anti-snake venom serum at home and abroad, the initial dose of 2–4 branches is reasonable and effective. Without medical insurance payment, the treatment cost for snake bites accounts for a large part of the annual net income. We speculate that it may cause a huge economic burden to the family. This survey lacks statistics on hospitalization costs and the average annual income of patients, which is also an indication of the limitations of this article. It will also be listed as a research plan for our team in the future.

Standardized treatment protocols involve the controlled administration of antivenom serum and the management of complications associated with venomous snakebites (Jeon et al., 2019). The purpose of debridement is to identify and remove any broken teeth, cleanse the contaminated or infected wound surface, and remove local necrotic tissue. Incisional debridement should only be considered when compartment syndrome is suspected, despite adequate administration of antivenom serum (Navaeifar et al., 2023; Canas, 2024). When conditions permit, negative pressure suction to remove local snake venom can also be used. One commonly used method is local vacuum sealing drainage (VSD) (Luo et al., 2020), which can promote wound healing (Normandin et al., 2021). However, studies have shown that VSD is not superior to standard treatment and may even cause complications such as toxic shock syndrome, infection, pain, bleeding, ischemia of adjacent tissues, and even hemodynamic instability (Toschlog et al., 2013). Unregulated debridement or incisions should be avoided to reduce the risk of complications (Mao et al., 2021). While tetanus is rarely a complication of venomous snakebites, the small fang marks and the potential presence of *Clostridium* bacteria in the snake’s oral cavity still pose a risk for tetanus infection (Di Nicola et al., 2021). Therefore, routine tetanus prophylaxis is recommended following snakebites. Moreover, treatments such as local hormone application for pain relief and wet compresses with magnesium sulfate for reducing swelling can be considered, although their clinical effects require further validation. This research discloses that in Yunnan Province, venomous snakebites are predominantly of hemotoxic type (Faisal et al., 2021), which can lead to VICC or active bleeding (Si et al., 2014; Maduwage and Isbister, 2014). Some studies suggest that supplementing with coagulation factors, including plasma transfusion and fibrinogen, may improve outcomes (Holla et al., 2018; Qian and Ma, 1991). Tranexamic acid may also be considered based on thromboelastography (TEG) data (Abraham et al., 2020). JiDesheng snake pill (JDS) is a widely used traditional Chinese medicine, JDS is the most widely used and mainly contains Paris polyphylla Sm (qì yè yí zhīhuā), Toad Skin (chán chú pí), Centipede (wú gōng), and Euphorbia humifusa Willd (dì jíng cǎo). Currently, it has achieved excellent clinical treatment results in the field of venomous snake bites (Luo et al., 2024).

In the study, correlations between various treatment measures, including incision, debridement, local blockade, additional antivenom serum therapy, tetanus immunoglobulin, plasma and fibrinogen transfusions, tranexamic acid administration, JiDesheng Snake Medicine treatment, and magnesium sulfate wet compresses, and the duration of hospital stay were analyzed. The results indicated that standardized incision, debridement, supplementary antivenom serum, plasma transfusion, and fibrinogen transfusion were associated with a shorter recovery time and earlier discharge from the hospital. Plasma infusion is significantly associated with prolonged hospital stay. It is not clear whether this is due to the treatment itself or because more severe cases require such treatment. More research evidence is needed in the future to support this. In the 2024 Chinese Guidelines for the Treatment of snake bites, it is clearly stated that the purpose of wound debridement in venomous snakebite cases is to identify and remove any remaining broken teeth, clean the wound contamination or infection sites, and remove local necrotic tissue. The majority of venomous snakebite cases do not require debridement. For venomous snakebite cases accompanied by VICC, fresh frozen plasma or cryoprecipitate should not be routinely used as blood products, but for severe coagulation dysfunction requiring emergency surgery, invasive procedures, or when using adequate antivenom serum still fails to improve the condition or there is active bleeding, it can be considered as an adjunctive therapy on the basis of adequate antivenom serum (Lai et al., 2024). Therefore, the timing of debridement treatment and the use of fibrinogen is limited and needs to be analyzed specifically based on clinical circumstances. However, tranexamic acid did not show any benefit in shortening hospital stay in our study, and this result is consistent with the results of related studies. The therapeutic value of tranexamic acid requires further research (Abraham et al., 2020).

To summarize, snakebite incidents in Yunnan Province mainly occurred from June to September. The genera *Ovophis* and *Trimeresurus* were most commonly associated with these bites, followed by *Agkistrodon*, *Vipera*, *Naja*, *Anilius*, and *Ophiophagus*. Bites were predominantly reported in paddy fields or mountainous terrains, most affecting the limbs. Typical clinical manifestations included pain and swelling in the limbs, and the most common complication was VICC. The early and proper administration of antivenoms and standardized treatment protocols are critical for effectively managing snakebite cases. In light of these epidemiological patterns, a collaborative research group focused on snakebites was established in Yunnan Province in 2024. The group aims to create diagnostic and treatment research centers in high-risk areas throughout all prefectures and cities in the province. The group intends to develop locally appropriate snakebite treatment and prevention guidelines by conducting multi-center prospective studies. Moreover, the initiative will strengthen scientific research on snakebites, improve basic treatment knowledge and training for populations in remote rural regions, expand treatment access in high-risk areas, elevate the treatment standards at grassroots medical institutions, and optimize the availability of antivenom reserves in local hospitals. Ultimately, the project intends to reduce the socio-economic burden of snakebites and minimize their impact on public health. This study is also subject to certain limitations. Primarily, the incomplete data reported by some prefectures and cities in Yunnan Province may result in an underestimation of the overall situation of venomous snakebite incidents in Yunnan Province; it may potentially impact the analysis of the influencing factors related to venomous snakebites. In the subsequent step, we will intensify the supervision and guidance over the data reporting work on venomous snakebites in various prefectures and cities to guarantee the completeness and accuracy of the data; establish a well-rounded monitoring system for venomous snakebites to enhance the efficiency and quality of data collection and reporting. This will increase the accuracy and reliability of the research and provide vigorous support for formulating more effective policies for the prevention and treatment of venomous snakebites.

## 5 Conclusion

This research emphasizes the importance of addressing venomous snakebites as a pressing public health concern, with an urgent need for gathering comprehensive epidemiological data across various regions. Hemotoxic snakebites are particularly prevalent in Yunnan Province. Establishing warning systems in areas and seasons where snake activity is frequent is crucial and providing specialized training and personal protective equipment for individuals in high-risk occupations is crucial. Furthermore, essential measures include establishing emergency treatment stations in areas with high snakebite rates, ensuring sufficient antivenom availability, establish a well-developed data reporting system for snakebite cases, and improving medical staff’s competency in snakebite management protocols.

## Data Availability

The original contributions presented in the study are included in the article/supplementary material, further inquiries can be directed to the corresponding authors.
